# Neuropsychiatric effects of Synthetic Cathinones - a review

**DOI:** 10.1192/j.eurpsy.2023.1379

**Published:** 2023-07-19

**Authors:** J. Mendes Coelho, M. Bicho, C. Peixoto, H. Fontes

**Affiliations:** Psychiatry, Hospital do Divino Espírito Santo de Ponta Delgada, Ponta Delgada, Portugal

## Abstract

**Introduction:**

The emergence of new psychoactive substances (NPS) has had a substantial impact worldwide. NPS mimic the effect of “existing” drugs and are specifically manufactured so that the new substances fall out of regulatory frameworks. Although the structural changes might be minimal, NPS often have marked differences in potency and subsequent harm.

The population of the Azores archipelago has been particularly affected by the rapid growing and changing nature of this phenomenon, mainly caused by the introduction and spread of the “newly” synthetic cathinones as a inexpensive and easily available street drug.

Before any educational, public health and socioeconomic policy changes be proposed to accurately tackle the problem, the basic step of knowing how these substances have been affecting their users, specially their neuropsychiatric effects, must be taken.

**Objectives:**

This project aims to characterize the neuropsychiatric side effects caused by the acute intoxication of NPS with a psychostimulant profile, namely synthetic cathinones.

**Methods:**

Description of the neuropsychiatric symptoms of intoxicated users of stimulant NPS that present to non-governmental organizations specialized in addictions and also to the psychiatry emergency department in Sao Miguel - Azores.

Review of the forensic records of the deaths by suicide in 2021 in Sao Miguel, looking for evidence of recent abuse of NPS or previous history of “NPS use disorder”.

Non-systematic review of the recent and relevant scientific literature on this topic.

**Results:**

The desired effects are increased energy, mood enhancement, euphoria, mental clarity, improved concentration, improved sociability, increased talkativeness, empathy inducing effects, amplification of sound and colour and prosexual effects. Nevertheless, in the case of intoxication, the frequent neuropsychiatric side effects tend to be agitation, agressiveness, irritability, altered consciousness, brief psychosis with paranoid delusion, visual and auditive hallucinations, transient mania, enhanced sensorial experiences, headaches, dizzyness, seizures, confusion and amnesia. Usually, the acjute intoxication period tend to be followed by a “crash” with depression, craving, anxiety, panic, suicidal ideation and behaviours. A third of the deaths by suicides in Sao Miguel Island in 2021, 7 out of 21, were in stimulant NPS active or recent users.

**Conclusions:**

Review of the neuropsychiatric effects of New Psychoactive Substances with a psychostimulant profile. Further studies of this population of synthetic cathinone users in the Azores are due, namely studying their socioeconomic background, looking for risk and protective factors, and also the long-term side effects.

**Disclosure of Interest:**

None Declared

